# Association between Hyperactivity and SSB Consumption in Schoolchildren: A Cross-Sectional Study in China

**DOI:** 10.3390/nu15041034

**Published:** 2023-02-19

**Authors:** Yushan Zhang, Zhaohuan Gui, Nan Jiang, Xueya Pu, Meiling Liu, Yingqi Pu, Shan Huang, Shaoyi Huang, Yajun Chen

**Affiliations:** Department of Maternal and Child Health, School of Public Health, Sun Yat-sen University, Guangzhou 510080, China

**Keywords:** sugar-sweetened beverages, attention-deficit/hyperactivity disorder, children

## Abstract

Sugar-sweetened beverages (SSBs) consumption has risen significantly, which may lead to various health problems. Studies about the association between SSBs and attention-deficit/hyperactivity disorder (ADHD) in children are rare and inconsistent. We have used the two-stage cluster sampling method to select 6541 students aged 6–12. We further investigated their basic information and SSB intake. Teachers’ questionnaires and parents’ questionnaires were used to evaluating the hyperactive behaviors in children. We examined the associations between SSB consumption and hyperactivity index (HI) by adopting the censored least absolute deviation (CLAD) estimator. Then, we further evaluated the impacts of sex and age on the association between SSB intake and hyperactivity. Children who weekly drank SSB two or more times were associated with 0.05 (0.04, 0.07) and 0.04 (0.02, 0.06) higher scores of ln (HI+1) reported by teachers and parents, respectively, compared to non-consumers children (*p* for trend < 0.05). A stronger association between SSB intake and hyperactivity occurred in girls and old children. (*p* for interaction < 0.05). SSB intake has a positive correlation with the risk of hyperactivity in children, and the frequency of SSB consumption and hyperactivity have a dose–response relationship.

## 1. Introduction

Sucrose, also known as refined sugar, is a carbohydrate that can be rapidly metabolized by the human body. It is also a widely commercialized product that is often added to food [1], which contributes to excess energy intake in the human body. High-caloric sweeteners with artificial additives such as sucrose, high-fructose corn syrup (HFCS), or fruit juice concentrates, referred to as SSB [2], including soft drinks, energy drinks, caffeinated drinks, and some juices, are the primary source of added sugars [3]. Data showed that SSB consumption in the whole population averages up to 8 ounces per day [4], accounting for 3% to 10% of the global average daily energy consumption [5,6,7,8]. The excessive consumption of SSB brings significant burdens on the population’s health problem, especially among children and adolescents, who are not only the core consumer group of SSB, but also at a critical stage of physical and mental development. Under the initiative of the WHO, amounts of countries have issued relative policies restricting SSB in recent years, but limited progress has been made [9,10]. The negative impact of SSB consumption in children and adolescents remains a significant public health problem. Numerous shreds of evidence have shown that SSB intake is not only related to obesity, hypertension, and type 2 diabetes mellitus (T2DM) [11,12,13,14,15], but also affects the development of children’s nervous system [16,17].

ADHD is a neurobehavioral disorder in children whose core symptoms include attention problems, hyperactivity, and impulsivity, usually accompanied by behavioral problems such as learning difficulties, conduct disorder, maladjustment, and so on [18,19]. In recent decades, the estimated global prevalence rate in children and adolescents has ranged from 5.29% [20] to 7.2% [21]. Studies proved that ADHD is affected by many factors. The heritability of ADHD is high, reaching 70% to 80% around, and environmental exposures have been proven to be associated with ADHD as well. As a changeable environmental factor, the dietary factor has been widely concerned. Current research on dietary aspects, mainly focused on dietary patterns, which are described by “Junk food,” “Processed food,” “Snack,” “Sweet,” “Mediterranean diet,” and “Western-like”, were found to affect ADHD in different studies [22]. However, the comparability between these studies is terrible, due to the definition of the dietary pattern being complex, and it is also challenging to determine the dietary part that produces an effect. Hence, as an essential part of children’s diet patterns, sugar received more attention. Some studies have shown that sugar intake is associated with an increased risk of ADHD, and the mechanism of this association is thought to be related to the reward system or IGF2 hypermethylation.

As the main source of added sugar for human consumption, SSB consumption can estimate the sugar intake relatively simply and accurately. However, there are studies rarely investigating the relationship between SSB consumption and ADHD in school-age children, and the conclusions are inconsistent [13,23,24,25,26,27]. A study found a weak positive correlation between maternal prenatal exposure to sweet carbonated beverages (SCB) and ADHD in offspring [28]. Another study found no correlation between sugar consumption and the incidence rate of ADHD in children aged 6–11 years [26]. In addition, most of these studies have a small sample size. In most studies, only parents’ or teachers’ questionnaires were used alone to evaluate children’s hyperactivity behavior, which may lead to subjective bias in the assessment results. For example, the hyperactivity behavior reported by teachers probably obtains a higher score than that reported by children’s parents.

Therefore, this study was based on a cross-sectional survey of children in Guangzhou, southern China. At the same time, two questionnaires, Conners Parent Rating Scale—Revised: Short form (CPRS-R:S) and Conners Teacher Rating Scale—Revised: Short form (CTRS-R:S), were used to assess children’s hyperactive behaviors and to explore the association between SSB consumption and children’s hyperactive behaviors.

## 2. Materials and Methods

### 2.1. Participants and Data Collection Sample Sources

Data for this study are derived from a cross-sectional survey project conducted in 2019 among students in Guangzhou, south China. In this project, the demographic information and dietary habit information of the participants were obtained through questionnaires, and the psychological and behavioral problems were evaluated. Participants were collected by two-stage cluster sampling. The details are as follows: first, we randomly selected three urban districts and two suburban districts; a total of five districts was chosen from Guangzhou. Second, we selected one primary school out of random selection in each district, and a total of five schools were chosen. Third, we invited all students from the 5 selected schools (a total of 8692 qualified participants) to participate in the project. Finally, 6883 children and parents agreed to participate (79.2% participation rate). We excluded 342 children who had no information on SSB, hyperactive behavior, or possibly important confounders, leaving 6541 participants aged 6–12 years old.

This study has been approved by the Biomedical Research Ethics Review Board of Sun Yat-sen University and registered in the Chinese Clinical Trial Registry at NCT03582709. Written consent was obtained from all parents/guardians of the children before data collection.

### 2.2. Sugar-Sweetened Beverage Consumption

The information on SSB consumption was obtained by questionnaire, which mainly consists of two questions for children’s parents. (i) “In the past seven days, how many times has your child consumed solid beverages such as cola, Sprite, fruit drinks (orange juice drinks, etc.), nutritious fast food, Red Bull?”. Parents who answered consumption over 0 times needed to answer the amount of SSB consumed each time, based on the following question: (ii) “On average, how much SSB did your child drink per session? (One serving is equivalent to 250 milliliters)”. A large number of participants drank SSB zero times, resulting in an imbalance in the data on the SSB consumption volume. Therefore, we have considered a new grouping scheme to classify the SSB consumption into 0 times per week, 1 time per week, and at least 2 times per week by counting the frequency of SSB consumption to ensure enough participants in each group. In addition of that, we also estimated the weekly SSB intake (intake/week) of each child by the times of SSB ingests per week multiplied by each intake [29].

### 2.3. Hyperactive Behavior

Children’s hyperactive behavior was evaluated using both parents’ and teachers’ reports of Conners’ Rating Scale—Revised: Short form (CPRS-R:S and CTRS-R:S), which were widely used in epidemiological and clinical studies [30,31] and validated in schoolchildren in China with acceptable internal consistency values of 0.93 and of 0.94, respectively. CPRS-R: S contains 27 questions, including cognitive problems/inattention, hyperactivity, opposition, and the ADHD index in 4 scales, which are used to evaluate children’s behavior problems. The CTRS-R:S contains 28 questions, including items on ADHD of 4 subscales and comorbid conditions in the school setting. Both questionnaires include the hyperactivity index (HI), which was summed from 10 items with a 4-point response scale (0 for “never,” 1 for “sometimes,” 2 for “often,” and 3 for “very often”). A higher value of HI is associated with more symptoms of hyperactivity. Children were classified as having clinically significant hyperactive symptoms as HI > 2SDs above the mean according to the age and sex standards.

### 2.4. Covariates

Some relative covariates were considered in the analysis. Information about socio-demographic factors and lifestyle was obtained through the questionnaire, which mainly contained: age (year), sex (male or female), single child (yes or no), monthly household income (≥12,000 RMB or refused to answer, 8000–11,999 RMB, 5000–7999 RMB, <5000 RMB), parent’s degree (university or above, college, senior high, below senior high), parental smoking status (never smoke, previously or presently smoked), and outdoor activity (>4 h/day, 2–4 h/day, 1–1.9 h/day, or <1 h/day). We have collected dietetic behavior information using the following three questions: “In the past 7 days, how many times did your child eat (1) deep-fry food; (2) fish or fish products; and (3) milk, or dairy foods?”. We measured the anthropometric data (height, weight) in children who were required to wear light clothes and be barefoot.

The body mass index (BMI) was calculated as BMI = weight (kg)/height (m)^2^ according to the World Health Organization’s recommendations.

### 2.5. Statistical Analyses

Continuous data were described as the mean and standard deviation (SD) and were compared using the analysis of variance (ANOVA). Categorical data were reported as numbers and percentages and were analyzed by using the analysis of a chi-square test.

We examined the associations between SSB consumption and HI (convert to the natural log of HI plus one [ln (HI score + 1)] to reduce skewness) by adopting the censored least absolute deviation (CLAD) estimator [31]. Then, we employed unconditional binary logistic regression models to assess the relationship between SSB consumption and the risk of children’s hyperactivity. We developed three models, including a crude, unadjusted model and two adjusted models, adding different covariates. Model 1 has controlled the following variables: sex, age, single child, monthly household income, parent’s degree, parental smoking status, outdoor activity, and body mass index. Model 2 has additionally controlled variables such as deep-fry food, fish or fish products, and milk or dairy foods. We performed trend tests in the three models. Then, we evaluated the dose–response relationship between the weekly intake of SSB and hyperactive behavior in children who consume SSB. In addition, in order to test potential effect modifications, several stratified analyses were completed, analyzed by sex (male, female) and age (<10 years, ≥10 years). In addition, the importance of interaction terms was examined.

Data analyses were conducted with Stata software version 16 (Stata Corp LLC., College Station, TX, USA). All *p*-values were two-tailed and the criteria for significance were *p*  <  0.05.

## 3. Results

### 3.1. Baseline Characteristics

The mean (SD) age of the 6541 participants was 8.6 (1.5) years old; 3502 (53.5%) of which were boys. A total of 4224 children (64.6%) had SSB consumption, of which 1958 children (29.9%) consumed SSB once a week and 2266 children (34.6%) consumed SSB more than twice a week (Table 1). The average amount of SSB consumed by participants per week was 2.45 (2.57). Consumers of SSB were significantly older, boys, had parental smokers, a higher BMI, and have a higher intake of levels of deep-fry foods, fish or fish products, and milk or dairy foods compared with non-consumers (All *p* values < 0.05).

### 3.2. Association between SSB Consumption and Hyperactive Behavior

SSB consumption was related to inferior performance of the hyperactive index (Table 2). In adjusted model 1, the teacher-reported ln (HI+1) scores were 0.05 (0.32, 0.72) and 0.07 (0.05, 0.08) higher, and the parent-reported ln (HI+1) scores were 0.02 (0.01, 0.04) and 0.06 (0.04, 0.08) higher, for children who consumed SSB once per week and those who consumed SSB twice or more per week, respectively, compared with those who never consumed SSB. In adjusted model 2, the teacher-reported ln (HI+1) scores were 0.04 (0.02, 0.05) and 0.05 (0.04, 0.07) higher, and the parent-reported ln (HI+1) scores were 0.02 (0.00, 0.04) and 0.04 (0.02, 0.06) higher for children who consumed SSB once per week and those who consumed SSB twice or more per week, respectively, compared with those who never consumed SSB (All *p* values < 0.05).

Table 3 presents crude and adjusted analyses on the association between SSB intake and hyperactive behaviors reported by children’s teachers and parents, respectively. No association was observed in either the crude model or the adjusted model. In Model 1, compared with children who did not consume SSB weekly, children who consumed SSB once a week had an OR, as described by their teachers and parents, of 1.13 (0.88, 1.46) and 1.19 (0.85, 1.66), respectively, while for children who consumed SSB ≥ 2 times per week, the OR, as described by their teachers and parents, was 1.11 (0.87, 1.42) and 1.23 (0.89, 1.70), respectively. Among the children who drunk SSB once a week, the OR in model 2 was 1.13 (0.88, 1.46) and 1.16 (0.83, 1.62), respectively, as described by teachers and parents. Among the children who drank SSB twice or more times a week, the OR in model 2 among teachers was 1.07 (0.82, 1.39) and, among parents, 1.17 (0.83, 1.66), compared with their peers who had no consumption (this was taken as the reference).

### 3.3. Effect Modification

We further analyzed the impact of age and gender on the association between SSB and ADHD. We found that age and sex modified the association between SSB consumption with the hyperactive index reported by teachers and parents. The association between SSB intake and hyperactivity was more substantial in girls and older children (Table 4). Among female children, children who consumed SSB once a week and those who consumed SSB ≥2 times a week, compared with children who did not consume SSB weekly, ln the (HI+1) scores reported by their teachers were 0.10 (0.069, 0.130) and 0.12 (0.085, 0.150) higher, respectively (*p* for interaction < 0.05). In children aged 10 years and older, children who consumed SSB once a week and those who consumed SSB ≥2 times a week, compared with children who did not consume SSB weekly, ln the (HI+1) scores reported by their teachers were 0.06 (0.039, 0.089) and 0.06 (0.039, 0.089) higher, respectively, and the ln (HI+1) scores reported by their parents were 0.06 (0.029, 0.097) and 0.11 (0.076, 0.140) higher, respectively (*p* for interaction < 0.05).

## 4. Discussion

The present study examined the association between SSB consumption and hyperactivity among schoolchildren in Guangzhou, China. The current findings demonstrate that SSB consumption was significantly associated with hyperactive behavior, and the association remained robust after adjusted covariates of socio-demographic factors, lifestyles, and diets in Chinese children. In addition, age and sex may be potential influencing factors for these associations. The association between SSB intake and hyperactivity was more substantial in girls and older children.

In this cross-sectional analysis of 6541 children aged 6 to 12, 64.4% consumed SSB at least once a week, comparable to rates in the United States (67.2%) [6], while this is lower than the rate in Wales (74%) [17]. To the best of our knowledge, several studies have examined the association between SSB and hyperactivity behavior in children, most of which yielded results consistent with ours [32,33,34]. A cross-sectional study based on children aged 6–12 years in Korea (*n* = 16,831) assessed ADHD symptoms in children by asking their parents to complete the ADHD Rating Scale (K-ARS) and showed that soft drink intake was positively associated with K-ARS scores in children [32]. Schwartz DL et al. conducted a health behavior survey of 1649 middle school students in grades 7, 8, and 12 at 5 schools and found that the risk of ADHD increased by 14% for each additional SSB consumed after adjusting for multiple covariates such as age, race/ethnicity, and gender. Additionally, it found that students who consumed energy drinks had a 66% increased risk of hyperactivity/inattention after adjusting the consumption amounts of drinks, drinking other types of beverages, and other potential confounding factors [33]. A case–control study conducted in Taiwan among children aged 4 to 15 (case = 173; control = 159) reported that compared with those who did not consume SSB, children with moderate and high levels of SSB had 1.36 and 3.69 risks of ADHD, respectively (*p* < 0.05) [34]. In contrast, several studies have yielded inconsistent results [13,26]. Recently, Geng et al. conducted a cross-sectional survey of behavioral problems in children aged 3–6 years from 11 kindergartners in 109 cities in China (*n* = 27,987), and the results showed that there was no significant association between high SSB intake and ADHD, and no gender differences were observed [13]. Similarly, the results from another study, the Pelotas 2004 Birth Cohort Study in Brazil, showed a positive association between sugar intake and ADHD when they performed a cross-sectional analysis of children aged 6 years. However, the longitudinal analysis found that changes in sugar intake in children aged 6 to 11 years did not have an effect on ADHD incidence [26]. These inconsistent results may be due to various factors in the studies. These factors included heterogeneity of the population for study (e.g., age, genetic background, and lifestyle), different methods for estimating SSB consumption, different methods of measuring hyperactivity behavior in children, and inconsistent selection of confounding variables in data analysis. In conclusion, our findings supported a positive association between SSB consumption and hyperactivity behavior in children. However, the influence of each component in SSB should be further studied.

We also found that sex and age influenced the relationship between SSB consumption and hyperactive behavior in children. In the subgroup of female children, the association between SSB intake, reported by their teachers, and hyperactivity was stronger. This result may be caused by genetic polymorphisms in different genders. Previous studies have shown that sex differences are more obvious in individuals with mental development disorders [35]. Some studies have shown that males diagnosed with ADHD are more likely to accept the diagnosis of oppositional defiance disorder or behavioral disorder, while females are more likely to experience emotional regulation problems [36,37]. However, several studies have shown inconsistent results with our study in regard to how boys are more likely to exhibit symptoms of ADHD [38,39,40]. This may be due to recognition bias. Teachers and parents usually rated boys’ symptoms higher than girls’, even though there was no difference in their behavior. For the sex subgroup, parents’ preference for their children might be an explain to the difference between teachers’ and parents’ reports. We also found that in the subgroup of children ≥10 years old, the association between SSB intake and hyperactivity, whether reported by teachers or parents, was stronger. Previous data have shown that adolescents have the highest consumption of SSB [41]. In addition, children who consume SSB early are more likely to continue consumption [42]. Moreover, it is challenging to recognize ADHD symptoms in early childhood, which may also cause diagnostic bias.

There are several possible mechanisms to explain the connection between SSB consumption and ADHD. One possible mechanism may be related to the reward systems. Schwartz et al. reported that sugar consumption might involve a higher release of extracellular dopamine [43]. Therefore, prolonged sugar intake may cause desensitization of dopaminergic receptors. In order to achieve the same satisfaction, increased sugar intake is required, resulting in a gradual decrease in the dopamine response. This dysfunction of dopaminergic signaling will promote the inhibition of control mechanisms in the frontal cortex, a region directly related to the neurobiology of ADHD [44]. Another possible mechanism is that high-sugar foods with a low free choline content may cause hypermethylation of IGF2, which affects the development of ADHD symptoms in adolescents with EOP behavioral problems [45]. The high-sugar diet may also alter the human gut microbiome through the microbiome–gut–brain axis, and then modify some central nervous system receptors to influence the brain function as well as to exert epigenetic control of the gene expression, leading to behavioral problems [46].

There are some strengths in this study. First, we used both parents’ and teachers’ questionnaires to assess children’s hyperactive behavior. The bias from the single population estimate has been controlled. Second, we fully accounted for potential covariates and performed subgroup analyses. However, there were some limits in this study. First, the cross-sectional study design cannot infer causality relationship between social security consumption and the risk of hyperactivity behavior in children, and future prospective cohort studies or randomized controlled studies are needed to determine this. Second, the obtained SSB consumption was not precise enough, which may have led to the misclassification of some exposures. Third, we did not measure other sugars derived from high-sugar foods, potentially leading to a classification bias in SSB consumption. Fourth, recall bias and information bias from Social Security consumption assessments, parental ratings of hyperactivity behavior, and questionnaire-based socio-demographic factors may be unavoidable. Fifth, despite careful adjustment for a wide range of covariates in the model, residual confounding due to the unavailability of data may exist.

## 5. Conclusions

In summary, our findings suggested a positive association between SSB consumption and the risk of hyperactivity in children and a dose–response relationship between the frequency of SSB consumption and the risk of hyperactivity behavior. The problem of the excessive intake of SSB is widespread in many countries, and this finding has important implications for guiding policymakers in implementing intervention strategies. However, given the limitations of our study, more well-designed longitudinal studies are needed to confirm these findings.

## Figures and Tables

**Table 1:** Baseline characteristics according to SSB intake.

Characteristics	Total Sample	SSB Intake			*p* Value
		0 Time/Week	1 Time/Week	≥2 Times/Week	
n	6541	2317 (35.4)	1958 (29.9)	2266 (34.6)	
Age (years)	8.6 ± 1.5	8.4 ± 1.5	8.5 ± 1.5	8.8 ± 1.5	<0.0001
Sex					<0.0001
Male	3502 (53.5)	1146 (32.7)	1048 (29.9)	1308 (37.4)	
Female	3039 (46.5)	1171 (38.5)	910 (29.9)	958 (31.5)	
Single child					0.217
Yes	2751 (42.2)	943 (34.3)	827 (30.1)	981 (35.7)	
No	3770 (57.8)	1363 (36.2)	1130 (30.0)	1277 (33.9)	
Monthly household income					0.033
<5000 RMB	870 (13.3)	304 (34.9)	264 (30.3)	302 (34.7)	
5000–7999 RMB	1409 (21.6)	490 (34.8)	422 (30.0)	497 (35.3)	
8000–11,999 RMB	1330 (20.4)	436 (32.8)	392 (29.5)	502 (37.7)	
>12,000 RMB	2290 (35.1)	826 (36.1)	712 (31.1)	752 (33.0)	
Refused to answer	622 (9.5)	250 (40.2)	167 (26.9)	205 (33.0)	
Parent’s degree					0.378
Below senior high school	192 (2.9)	65 (33.9)	57 (29.7)	70 (36.5)	
Senior high school	684 (10.5)	237 (34.7)	186 (27.2)	261 (38.2)	
College	1199 (18.4)	413 (34.5)	377 (31.4)	409 (34.1)	
University or above	4446 (68.2)	1591 (35.8)	1337 (30.1)	1518 (34.1)	
Parental smoking status					<0.0001
Never smoke	3813 (58.5)	1457 (38.2)	1145 (30.0)	1211 (31.8)	
Previously smoked	764 (11.7)	239 (31.3)	223 (29.2)	302 (39.5)	
Presently smoked	1944 (29.8)	610 (31.4)	589 (30.3)	745 (38.3)	
Outdoor activity					0.879
<1 h/day	2814 (43.2)	979 (34.8)	863 (30.7)	972 (34.5)	
1–1.9 h/day	2866 (44.0)	1021 (35.6)	851 (29.7)	994 (34.7)	
2–4 h/day	627 (9.6)	223 (35.6)	181 (28.9)	223 (35.6)	
>4 h/day	214 (3.3)	83 (38.8)	62 (29.0)	69 (32.2)	
BMI (kg/m^2^)	16.8 ± 3.0	16.6 ± 3.0	16.7 ± 2.9	17.1 ± 3.1	<0.0001
Deep-fry food (times/week)	0.8 ± 1.0	0.4 ± 0.8	0.8 ± 0.8	1.2 ± 1.2	<0.0001
Fish or fish products (times/week)	3.2 ± 2.6	2.9 ± 2.1	2.9 ± 2.0	3.1 ± 2.1	<0.001
Milk or dairy foods (times/week)	6.7 ± 7.3	6.5 ± 3.3	6.3 ± 3.3	6.6 ± 3.2	0.016

Note: Continuous variables are expressed as mean (standard deviation) and categorical variables are expressed as number (percentage). BMI, abbreviation for body mass index; SSB, abbreviation for sugar-sweetened beverages.

**Table 2:** Association between SSB with hyperactive index.

Hyperactive Behavior (*n* = 6541)	Estimates (95% Confidence Interval)		
	Crude Model	Model 1 ^a^	Model 2 ^b^
Teacher report of HI			
0 time/week	Reference	Reference	Reference
1 time/week	0.09 (−0.11, 0.28) ^c^	0.05 (0.32, 0.72) ^c^	0.04 (0.02, 0.05) ^c^
≥2 times/week	0.09 (−0.10, 0.28) ^c^	0.07 (0.05, 0.08) ^c^	0.05 (0.04, 0.07) ^c^
*p* for trend	0.082	<0.0001	0.013
Parent report of HI			
0 time/week	Reference	Reference	Reference
1 time/week	0.07 (−0.09, 0.24) ^c^	0.02 (0.01, 0.04) ^c^	0.02 (0.00, 0.04) ^c^
≥2 times/week	0.07 (−0.09, 0.23) ^c^	0.06 (0.04, 0.08) ^c^	0.04 (0.02, 0.06) ^c^
*p* for trend	0.078	<0.0001	<0.001

Note: ^a^ adjusted for sex, age, single child, monthly household income, parent’s degree, parental smoking status, outdoor activity, and body mass index. ^b^ Additionally adjusted for deep-fry food, fish or fish products, and milk or dairy foods. ^c^ Statistically significant association (*p* value < 0.05). HI, abbreviation for hyperactivity index.

**Table 3:** Association between SSB intake with hyperactivity.

Hyperactive Behaviors (*n* = 6541)	Odds Ratio (95% Confidence Interval)		
	Crude Model	Model 1 ^a^	Model 2 ^b^
Teacher report of hyperactivity			
0 time/week	Reference	Reference	Reference
1 time/week	1.11 (0.88, 1.41)	1.13 (0.88, 1.46)	1.13 (0.88, 1.46)
≥2 times/week	1.07 (0.85, 1.34)	1.11 (0.87, 1.42)	1.07 (0.82, 1.39)
*p* for trend	0.609	0.417	0.619
Parent report of hyperactivity			
0 time/week	Reference	Reference	Reference
1 time/week	1.15 (0.84, 1.58)	1.19 (0.85, 1.66)	1.16 (0.83, 1.62)
≥2 times/week	1.51 (0.85, 1.57)	1.23 (0.89, 1.70)	1.17 (0.83, 1.66)
*p* for trend	0.396	0.224	0.368

Note: ^a^ adjusted for sex, age, single child, monthly household income, parent’s degree, parental smoking status, outdoor activity, and body mass index. ^b^ Additionally adjusted for deep-fry food, fish or fish products, and milk or dairy foods.

**Table 4:** Subgroup analysis of association between hyperactive index and SSB consumption.

Subgroups	*n* (%)	Estimates (95% Confidence Interval)			*p* for Interaction
		0 Time/Week	1 Time/Week	≥2 Times/Week	
Teacher report of HI					
Sex					0.018
Male	3502 (53.5)	Reference	0.04 (−0.004, 0.075)	0.05 (0.013, 0.094)	
Female	3039 (46.5)	Reference	0.10 (0.069, 0.130)	0.12 (0.085, 0.150)	
Age (years)					0.004
<10	4460 (68.2)	Reference	0.03 (−0.009, 0.067)	0.06 (0.023, 0.106)	
≥10	2081 (31.8)	Reference	0.06 (0.039, 0.089)	0.06 (0.039, 0.089)	
Parent report of HI					
Sex					0.167
Male	3502 (53.5)	Reference	0.01 (−0.006, 0.033)	0.08 (0.056, 0.096)	
Female	3039 (46.5)	Reference	0.03 (0.015, 0.054)	0.05 (0.031, 0.070)	
Age (years)					<0.0001
<10	4460 (68.2)	Reference	0.03 (0.001, 0.054)	0.02 (−0.006, 0.050)	
≥10	2081 (31.8)	Reference	0.06 (0.029, 0.097)	0.11 (0.076, 0.140)	

## Data Availability

Because the participants refused to share their data, the data of this study could not be publicly obtained. The corresponding author has the right to provide the data obtained in this study.
